# Validation of an enzyme immunoassay for the detection of corticosterone metabolites from northern bobwhite (*Colinus virginianus*) feces

**DOI:** 10.1093/conphys/coad098

**Published:** 2024-01-28

**Authors:** Jeremiah Leach, Hannah N Suber, Katelyn Conley, Regan Rivera, James Surles, Benjamin Hames, Ronald J Kendall

**Affiliations:** Wildlife Toxicology Laboratory, Texas Tech University, 1234 Davis Drive, Lubbock, TX, USA; Wildlife Toxicology Laboratory, Texas Tech University, 1234 Davis Drive, Lubbock, TX, USA; Wildlife Toxicology Laboratory, Texas Tech University, 1234 Davis Drive, Lubbock, TX, USA; Wildlife Toxicology Laboratory, Texas Tech University, 1234 Davis Drive, Lubbock, TX, USA; Department of Mathematics and Statistics, Texas Tech University, 1108 Memorial Circle, Lubbock, TX 79409, USA; Wildlife Toxicology Laboratory, Texas Tech University, 1234 Davis Drive, Lubbock, TX, USA; Wildlife Toxicology Laboratory, Texas Tech University, 1234 Davis Drive, Lubbock, TX, USA

**Keywords:** *Colinus virginianus*, enzyme immunoassay, fecal corticosterone metabolites, northern bobwhite, stress

## Abstract

Interest in the effects of stressors on wildlife has grown substantially over the past few decades. As this interest has grown, so has the need for minimally invasive and reliable methods for estimating differences in the levels of stress hormones. An enzyme immunoassay using standardized methods was validated for detecting concentrations of corticosterone (cort) metabolites from northern bobwhite fecal samples. Two physiological challenges and one biological challenge were applied to 18 northern bobwhites (nine males and nine females), and the fecal cort metabolite concentrations were compared to baseline levels. The interactions of sex and treatment, treatment and time and sex and time were all significant. Thus, the methods and tools used here were sensitive enough to detect expected changes to the hypothalamo–pituitary–adrenal axis of northern bobwhite.

## Teaser

Measuring hormones in feces is an effective way to estimate stress levels in vertebrates. However, due to differences between species and sex, any method that uses fecal samples to estimate hormone levels must be validated. Here we validated an enzyme immunoassay to measure stress hormones in northern bobwhite feces.

## Introduction

Vertebrates maintain homeostasis through several mechanisms. An important mechanism is the hypothalamo–pituitary–adrenal (HPA) axis. Corticotropin-releasing hormone (CRH) is released from the hypothalamus in response to external and internal stimuli. CRH then triggers the pituitary to release adrenocorticotropic hormone (ACTH) into the bloodstream. With most vertebrates, ACTH stimulates the adrenal cortex to begin releasing and producing cortisol; however, in birds and rodents, corticosterone (cort) is produced. Cort then interacts with either the mineralocorticoid receptor (MR) or the glucocorticoid receptor (GR). Both MR and GR are nuclear receptors that, when activated, dimerize to upregulate the expression of several genes involved in maintaining homeostasis, including energy metabolism (Blas, 2015). Alterations of cort levels to acute stressors are usually, and expectedly so, beneficial to the individual. The behavioral and metabolic changes elicited by the increase in cort are expected to confer higher fitness than baseline cort in the presence of the stressor. Research into the HPA axis of wildlife is typically done in the context of allostasis. Allostasis is a model for describing changes in hormone levels for the maintenance of homeostasis (McEwen and Wingfield, 2003). Allostasis predicts that individuals naturally alter glucocorticoid levels in response to predictable events, but unpredictable events will cause alterations outside of the normal range (Wingfield, 1997; McEwen and Wingfield, 2003). A substantial alteration could cause stress hormone levels to exceed a threshold where individuals can no longer obtain sufficient energy to sustain demands. When this threshold is crossed, individuals are considered to be in an emergency life-history stage (ELHS) (McEwen and Wingfield, 2003). Repeated entry or prolonged periods of ELHS could lead to pathological consequences and reductions in overall fitness (McEwen and Wingfield, 2003; Blas, 2015).

There has been growing interest in evaluating differences in the HPA axis response to a variety of stressors. For example, Bokony *et al.* (2009) conducted a systematic review and found species with higher brood values (that is the value of a single brood is greater relative to other species) had a lower stress response compared to species with a lower brood value. This suggests differences in the tolerance between species to stressors, at least in the context of the HPA axis. Work done in tree swallows (*Tachycineta bicolor*) found that individuals exposed to chronic noise pollution did not habituate, had elevated baseline levels of cort and had lower nestling body condition compared to individuals not exposed to chronic noise pollution (Injaian *et al.,* 2018). There is also strong evidence that changes in the HPA axis can also alter the onset of breeding (Lattin *et al.,* 2016). The interactions of stress-induced elevated levels of cort and fitness measures are, however, quite complex (Wingfield *et al.,* 1992; Bokony *et al.,* 2009). Research into ruffed grouse (*Bonasa umbellus*) found that fecal cort metabolite (FCM) levels differed based on an individual’s roosting location and that FCM concentrations decreased as snow depth increased (Shipley *et al.,* 2019; Shipley *et al.,* 2021). Messina *et al.* (2020) found that feather cort level predicted relative abundance of several tropical bird species for the year following sampling, and the trend was stronger in logged forests compared to undisturbed forest areas. Research on black grouse (*Tetrao tetrix*) using FCM concentrations showed that disturbance by recreational winter activities could increase daily energy expenditure by as much as 10% (Arlettaz *et al.,* 2015). Despite this evidence, understanding the physiological consequences of stressors on wild birds, particularly North American game birds, remains an understudied area.

Northern bobwhite (*Colinus virginianus*) populations have declined appreciably over the past several decades (Hernández *et al.,* 2013). Habitat loss, degradation and climate change are likely the ultimate cause of the decline (Brennan, 1991; Hernández *et al.,* 2013); however, proximate factors such as pesticides, disease and anthropogenic disturbance are also likely contributors (Henry *et al.,* 2020; Moreau *et al.,* 2022). Little is known, however, about the physiological changes brought on by these factors in this species. One plausible reason for the discrepancy is the lack of accessible tools for accurately and reliably measuring changes in the HPA axis. An enzyme immunoassay (EIA) is one such tool for assessing changes in cort levels. Typically, cort is extracted from a blood sample, but this is not without its drawbacks. Foremost is the fact that the blood draw itself must be done quickly to avoid detecting the stress response to the capture or handling event instead of the factor the researcher is interested in (Cook, 2012). An alternative to measuring cort from blood samples is to measure FCM; however, a protocol for measuring FCM must first be validated (Palme, 2005; Touma and Palme, 2005). Thus, the purpose of this research was to validate an EIA for estimating stress hormones from bobwhite. The methods used were kept as simple as possible to increase the accessibility of the protocol and make it usable even under field conditions.

## Materials and Methods

### Animal care and ethics

A total of nine male and nine female bobwhites (all approximately 1 year old) were used for this study and maintained under Texas Tech University Institutional Animal Care and Use Committee protocol 2022-1178. Birds were housed individually, provided with food and water *ad libitum* [except during the biological (Bio) challenge] under a 12-h light–dark cycle.

### Determination of best solvent concentration

Fecal samples were collected by free catch over aluminum foil for 24 h from one male and one female bobwhite. The samples were pooled by each individual and stored at −20°C until further processing. Feces were dried by placing them in plastic weigh boats in a 14-l plastic latch box with four 112-g desiccation packs until completely dry (approximately 24 h). Once dried, the samples were homogenized by grinding them into a fine powder using a mortar and pestle. The mortar and pestle were cleaned using 70% ethanol (EtOH) between each homogenization. The samples from each bird were separated into 18 150-mg aliquots and placed in 2-ml microcentrifuge tubes. Six aliquots from both the male and female bobwhites were extracted using 60% EtOH, six using 70% EtOH and six using 80% EtOH. The extraction was done following Arbor Assays™ recommendations. Each extraction (750 μl) was placed in a clean 1.5-ml microcentrifuge tube, placed in a closable latch box with four 112-g desiccation packs, and allowed to dry down completely. Once dry, the samples were reconstituted with 50 μl of the appropriate EtOH solvent concentration. The samples were diluted using the assay buffer provided to a final EtOH concentration of 4% for use in the EIA. FCM was estimated using Arbor Assays™ DetectX® cort EIA kits following the manufacturer’s recommendations for the 100-μl format. The plate was analyzed using the Byonoy™ Absorbance 96® microplate reader at 450 nm. All three EtOH concentrations had similar intra-assay variation; however, the 60% EtOH solvent yielded the highest measurement and was used for the remainder of the validation.

### Treatment and sample collection

#### Baseline

Beginning at 8:00 h, individuals were removed from their cage and weighed. An aluminum foil sheet was placed under each cage to catch fresh droppings. Droppings were collected and pooled every hour for 9 h and placed in a freezer at −20°C immediately following the collection period. The baseline and each treatment were conducted 7 days apart.

#### Physiological challenge

Two physiological challenges were conducted during this validation: an ACTH and dexamethasone (DEX) challenge. Both challenges were administered by injecting the appropriate stimuli into the breast tissue using a 25-gauge insulin needle. ACTH (Sigma-Aldrich, ACTH Hormone Human, ≥97% purity) was administered at a dose of 120 μg/kg. This was shown to be the lowest effective dose for elevating cort levels in racing pigeons (Lumeij *et al.,* 1987). DEX (Sigma-Aldrich, DEX–water soluble) was administered at 0.75 mg/kg. Both ACTH and DEX were suspended in 0.9% sterile saline the morning of their respective challenges. Both challenges began at 0800 h. The birds were weighed, and the appropriate volume of ACTH or DEX was delivered. The birds were then returned to their cages, and feces were collected in the same manner as the baseline collections.

#### Bio challenge

The Bio challenge selected for this validation was food deprivation. Food deprivation was selected because it is a real stressor a bobwhite would be exposed to in nature. At 17:00 the day prior to fecal collection, food was removed from the study birds. At 8:00 the next morning, birds were removed from their cages, weighed and returned. Fecal samples were collected in the same manner as the baseline collection. Food was returned to the study birds at 17:00 on the day of sample collection.

### FCM extraction

Frozen samples were placed in 14-l latch boxes with at least four 112-g desiccation packs and allowed to dry completely. All samples were completely dry within 24 h. Once dry, the samples were pulverized and homogenized into a fine powder using a mortar and pestle. The fecal powder was weighed and when possible aliquoted into multiple samples of 100–150 mg. The average FCM concentration of the aliquots was used when a bird had multiple aliquots from a single time point. Extractions were done following the recommendations of the EIA manufacturer. EtOH (60%) was added to the sample at a ratio of 1 ml for every 100 mg of feces. The samples were then mixed for 30 min and centrifuged at 4°C for 15 min at 5000 rpm. The supernatant (500 μl or less) was transferred to a clean 1.5-ml microcentrifuge tube and placed in a 14-l latch box with at least four 112-g desiccation packs and allowed to dry completely. Once completely dry, the sample was stored at −20°C until the assay could be completed.

### EIA protocol

Arbor Assays™ DetectX® cort kit was used for the assay. The extracted samples were reconstituted with 50 μl of 60% EtOH and diluted with 750 μl of the provided assay buffer to get the EtOH concentration below 5%. The procedure was conducted according to the manufacturer’s instructions for the 50-μl format, and all samples were run in duplicate. The plate was analyzed using a Byonoy™ Absorbance 96® microplate reader at 450 nm using the four-parameter logistic curve function. Final concentrations were determined using Equation1, where *A* = assay concentration in picogram per milliliter, *D* = dilution factor, *R* = reconstitution factor and *E* = evaporation volume. Intra-assay variation was 5.58%, and inter-assay variation was 11.84%.

Equation 1. \begin{equation*} \left(\frac{A\ast D\ast R}{E}\right)/0.1\ \mathrm{g} \end{equation*}

### Parallelism check

Parallelism was verified using seven fecal samples. FCM was extracted using the above methods. Four stages of a 1:2 serial dilution of the extract were done (a total of five concentrations for each fecal sample) before the samples were dried down. FCM concentration estimates were log_2_ transformed and a linear regression was performed with dilution factor as the independent variable and log-transformed FCM concentration as dependent variable. The estimated slope of each regression and the standard curve were compared using an analysis of covariance (ANCOVA) with an interaction term for sample and dilution stage. The model used in the ANCOVA is shown in Equation2. The assumption of homoscedasticity was checked visually by plotting the residuals.

Equation 2. \begin{equation*} {\log}_2{y}_{ij}={\beta}_0+\left({\beta}_1+{\gamma}_i\right)j+{\varepsilon}_{ij} \end{equation*}

Using Equation2, each sample should produce a slope of −1. This was tested by removing the interaction term from Equation2, thus pooling all samples to generate a common slope. The common slope was then compared to −1.

### Analysis

A mixed-model analysis of variance (ANOVA) was performed using the log concentration of FCM as the dependent variable. The global model included individuals nested within sex, time, treatment and all interactions as predictor variables. The assumption of homoscedasticity was checked visually with a plot of the residuals. Tukey–Kramer test was performed to evaluate significant differences between each group. Analysis was done using SAS v. 9.4. The analysis for the parallelism check was done using MINITAB v 20.0.

## Results

### Parallelism check

**Figure 1:**
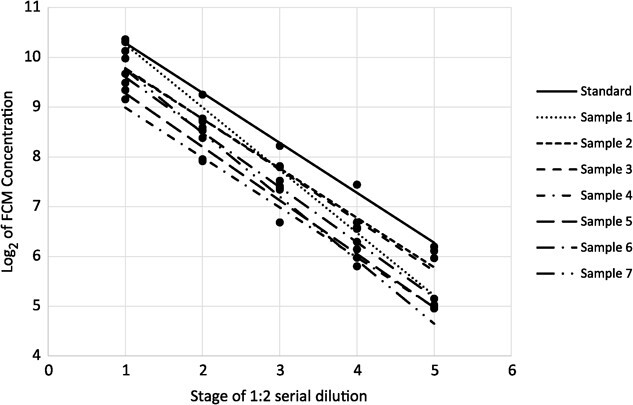
Regression of log_2_ transformed FCM concentrations from 7 unknown samples and the standard curve. Stage is the step in the serial dilutions with 1 being the undiluted sample.

The interaction term was non-significant (*P* = 0.128, *F*_7,38_ = 1.84). The estimate of the common slope was −1.0918 and was significantly different than −1 (*P* = 0.0129; 95% confidence interval, −1.021 to −1.163). Thus, the extraction protocol and assay are parallel to the standard curve, but the slope is not equal to −1. A plot of the data used in the parallelism check is shown in Fig. 1.

### Physiological and Bio challenges

**Figure 2:**
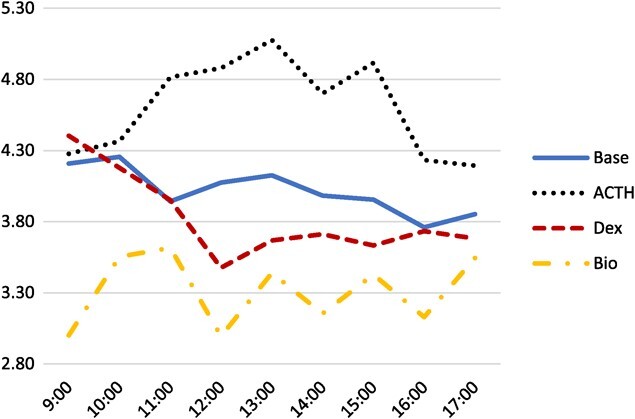
The interaction of treatment and time on mean log_10_ concentration of FCM extracted from male bobwhite feces and assayed on an EIA.

**Figure 3:**
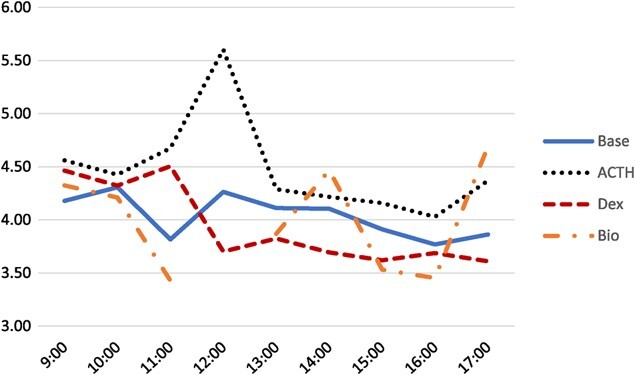
The interaction of treatment and time on mean log_10_ concentration of FCM extracted from female bobwhite feces and assayed on an EIA. There were no fecal samples to collect at the 12:00 time point.

A total of 474 samples were analyzed: 152 for the baseline, 128 for the ACTH challenge, 110 for the DEX challenge and 84 for the Bio challenge. A total of 22 EIA plates were used. The three-way interaction of sex, time and treatment was significant (*P* = 0.032); however, due to the unbalanced nature of the data, a post hoc analysis could not be performed. Additionally, the effect of the three-way interaction was minimal when considering the two-way interactions. Plots of the three-way interaction for males are displayed in Fig. 2 and for females in Fig. 3. The interaction of sex and treatment has the greatest effect (*F*_3,410_ = 15.97, *P* < 0.0001). The second greatest effect was the interaction of time and treatment (*F*_24,410_ = 3.74, *P* < 0.0001). Finally, the effect of the interaction of sex and time was the smallest (*F*_8,410_ = 2.13, *P* = 0.0322). Males and females show a similar response to both baseline and DEX levels, but males show a greater response to both ACTH and Bio treatments than females. Female and male ACTH was significantly different than all other pairwise groupings (*P* = 0.0011 and *P* < 0.0001, respectively). Male DEX and Bio were also significantly different than male base (*P* = 0.0485 and *P* < 0.0001, respectively). Neither the DEX (*P* = 0.5027) nor the Bio (*P* = 0.0516) treatments were significantly different in females; however, the female Bio treatment was nearly significant (Table 1).

**Table 1:** Least-square means and standard error for sex and treatment with pairwise groupings.

Sex	Treatment	Least-square mean (log concentration, pg/g)	SE (±)
Female	Base	3.96	0.037
	ACTH	**4.25 (*P* = 0.0011)**	0.067
	DEX	3.81 (*P* = 0.5027)	0.060
	BIO	3.70 (*P* = 0.0516)	0.140
Male	Base	3.98	0.075
	ACTH	**4.44 (*P* < 0.0001)**	0.061
	DEX	**3.77 (*P* = 0.0485)**	0.043
	BIO	**3.09 (*P* < 0.0001)**	0.076

Means in bold are statistically significant from baseline levels for the respective sex.

**Table 2:** Interactions between time and treatment with pairwise groupings.

Treatment	Time	Least-square mean (log concentration, pg/g)	SE (±)
Baseline	9:00	4.14	0.05
	10:00	4.22	0.05
	11:00	3.79	0.07
	12:00	4.12	0.06
	13:00	4.04	0.05
	14:00	4.02	0.05
	15:00	3.85	0.07
	16:00	3.72	0.06
	17:00	3.84	0.03
ACTH	9:00	4.12 (*P* = 1.0)	0.18
	10:00	4.21 (*P* = 1.0)	0.14
	11:00	**4.55 (*P* = 0.0002)**	0.15
	12:00	**4.85 (*P* = 0.0019)**	0.23
	13:00	4.49 (*P* = 0.6298)	0.18
	14:00	4.31 (*P* = 0.9785)	0.12
	15:00	4.33 (*P* = 0.1503)	0.10
	16:00	4.07 (*P* = 0.8017)	0.06
	17:00	4.19 (*P* = 0.7615)	0.06
DEX	9:00	4.36 (*P* = 1.0)	0.10
	10:00	4.18 (*P* = 1.0)	0.08
	11:00	4.02 (*P* = 1.0)	0.12
	12:00	**3.51 (*P* = 0.0278)**	0.08
	13:00	3.60 (*P* = 0.5543)	0.07
	14:00	3.63 (*P* = 0.7549)	0.07
	15:00	3.59 (*P* = 0.9997)	0.04
	16:00	3.62 (*P* = 1.0)	0.07
	17:00	3.61 (*P* = 0.9997)	0.06
Bio	9:00	**3.44 (*P* = 0.0033)**	0.25
	10:00	**3.46 (*P* = 0.0007)**	0.25
	11:00	3.34 (*P* = 0.9227)	0.16
	12:00	3.32 (*P* = 0.0970)	0.19
	13:00	3.54 (*P* = 0.4167)	0.12
	14:00	3.46 (*P* = 0.1420)	0.26
	15:00	3.18 (*P* = 0.0637)	0.23
	16:00	3.10 (*P* = 0.0508)	0.15
	17:00	3.68 (*P* = 1.0)	0.32

Least-square (LS) means in bold are significantly different than baseline at the same time point.

Table 2 shows the means and pairwise groupings of the time and treatment interaction. ACTHs at 11:00 and 12:00 were significantly different than the baseline measurements at those same times. The DEX treatment at 12:00 was significantly different than the baseline at 12:00 as well. Thus, concentrations of both physiological challenges deviated from the baseline concentrations, at the same time, before returning to a non-significant concentration from baseline concentrations. The Bio treatment was only significantly different than baseline levels at 9:00 and 10:00 but trended lower than baseline levels at all time points and was very similar to baseline levels at 5:00. The bio treatment was nearly significantly different than the baseline at 14:00 (*P* = 0.0508).

The significant *P* value from the sex and time interaction term indicates the two sexes differ across time in FCM excretions (Table 3). Females at 9:00 differ from females at 15:00 and 16:00 (*P* = 0.0197 and 0.001, respectively), but no other time points are significantly different within females. The only time points that were significantly different for males were the 11:00 and 16:00 time points (*P* = 0.0439). There were no significant differences between males and females at the same time point.

**Table 3:** LS means, SE and pairwise groupings of the interaction between sex and time.

Sex	Time	Least-square mean (log concentration, pg/g)	SE (±)
Female	9:00	**4.20**	0.09
	10:00	4.07	0.11
	11:00	3.86	0.14
	12:00	4.11	0.18
	13:00	3.90	0.08
	14:00	3.89	0.10
	15:00	**3.72 (*P* = 0.0197)**	0.08
	16:00	**3.67 (*P* = 0.0010)**	0.06
	17:00	3.95	0.10
Male	9:00	3.83	0.14
	10:00	3.96	0.11
	11:00	**3.99**	0.10
	12:00	3.79	0.14
	13:00	3.94	0.11
	14:00	3.81	0.11
	15:00	3.75	0.12
	16:00	**3.59 (*P* = 0.0439)**	0.09
	17:00	3.71	0.08

Means in bold indicate a significant difference with one or more means within the same sex. The *P* value listed for the 15:00 and 16:00 time points for females is the difference between those times and the 9:00 sampling period. The *P* value at the 16:00 sampling point is for the difference between the 11:00 sampling point.

## Discussion

Here we validated an EIA for detecting FCM from bobwhite feces. The peak in FCM following ACTH and DEX treatments was detected at the 12:00 sampling period, approximately 4 h following the respective injection. This is the expected time based on other research into measuring of FCM from bobwhite (Mohlman *et al.,* 2020) and similar to other avian species (Jepsen *et al.,* 2019). The FCM extraction and EIA protocol used here were able to detect expected changes in FCM in response to physiological challenges to the HPA. The Bio treatment was significantly lower than baseline in the males and was nearly significant in females. Significant differences were also detected across time for both sexes, and those differences were not the same between sexes. Thus, this protocol is a valid method for detecting changes to the HPA axis of bobwhite.

The significant interaction between sex and treatment was not unexpected (Touma and Palme, 2005); however, not all validated non-invasive assays have shown the same interaction (Mohlman *et al.,* 2020). Part of the reason the sexes responded differently to treatments may have been due to the timing of said treatments. Females were producing eggs throughout this experimental period and likely had a greater energy demand than males. Many studies have found that males and females respond differently to stressors during periods of egg laying, nesting and incubation (Wingfield *et al.,* 1992; Wingfield *et al.,* 1995). It is suspected that the difference in response is an adaptation to prevent unnecessary nest or brood abandonment, with the sex that serves as the primary caretaker showing a dampened response to stress induction (Wingfield *et al.,* 1995). Bobwhites do have a complex mating strategy, with about 10% to 36% of males tending nests (Cox *et al.,* 2005; Hernández *et al.,* 2007; Richardson *et al.,* 2020), but females are generally the ones that tend nests. Thus, the reduced response observed by females to all three treatments is likely due to adaptations to prevent unnecessary nest abandonment in response to an acute stressor.

Females and males did differ in FCM concentrations across the sampling periods; however, they did follow a similar trend. In both sexes, no sampling point was significantly different than the previous sampling point, but at least one before noon sample had a significantly different FCM than at least one afternoon sampling period. It is well established that plasma hormone levels fluctuate throughout the day, and cort levels are no exception. However, a key difference between cort concentrations in the plasma and cort metabolite concentrations in feces is they represent different sampling intervals (Palme, 2005; Cook, 2012). Plasma cort concentrations represent a point interval, which is the concentration at a specific time. This type of measurement is particularly useful when assessing acute stress or taking multiple measurements from an individual over time. FCM however represents the amount of free cort that was removed (and presumably used) from circulation, metabolized and secreted (Palme, 2005; Touma and Palme, 2005; Cook, 2012). Thus, FCM is representative of a time interval. As a result, measurements of plasma cort may be from peaks or troughs that occur in the amount of circulating cort, and FCM measurements represent an “average” over the interval. Thus, FCM measurements may not be suitable for detecting daily patterns of cort release. However, care should still be taken when using this methodology to compare cort levels between individuals. Individuals sampled within a few hours of each other may be comparable; individuals sampled in the morning may not be comparable to individuals sampled in the afternoon.

In conclusion, we biologically validated an EIA using simple extraction protocols that can be carried out under field conditions with limited equipment. This assay was able to detect physiological stimulation and suppression of the HPA axis as well as a biologically relevant stressor (food deprivation) in bobwhite. Furthermore, it detected changes in the HPA axis across the sampling period and differences between sex. Thus, the protocol used here is a valid method for estimating differences in the HPA axis of bobwhite.
